# Salecan Enhances the Activities of β-1,3-Glucanase and Decreases the Biomass of Soil-Borne Fungi

**DOI:** 10.1371/journal.pone.0134799

**Published:** 2015-08-06

**Authors:** Yunmei Chen, Haiyang Xu, Mengyi Zhou, Yang Wang, Shiming Wang, Jianfa Zhang

**Affiliations:** Center for Molecular Metabolism, Nanjing University of Science & Technology, Nanjing, China; Leibniz-Institute of Vegetable and Ornamental Crops, GERMANY

## Abstract

Salecan, a linear extracellular polysaccharide consisting of β-1,3-D-glucan, has potential applications in the food, pharmaceutical and cosmetic industries. The objective of this study was to evaluate the effects of salecan on soil microbial communities in a vegetable patch. Compositional shifts in the genetic structure of indigenous soil bacterial and fungal communities were monitored using culture-dependent dilution plating, culture-independent PCR-denaturing gradient gel electrophoresis (DGGE) and quantitative PCR. After 60 days, soil microorganism counts showed no significant variation in bacterial density and a marked decrease in the numbers of fungi. The DGGE profiles revealed that salecan changed the composition of the microbial community in soil by increasing the amount of *Bacillus* strains and decreasing the amount of *Fusarium* strains. Quantitative PCR confirmed that the populations of the soil-borne fungi *Fusarium oxysporum* and *Trichoderma* spp. were decreased approximately 6- and 2-fold, respectively, in soil containing salecan. This decrease in the amount of fungi can be explained by salecan inducing an increase in the activities of β-1,3-glucanase in the soil. These results suggest the promising application of salecan for biological control of pathogens of soil-borne fungi.

## Introduction

Soil management can significantly impact the biological and biochemical properties of soil; in particular, entry of a substrate, especially carbon, into the soil governs the activity and growth of the soil microbial biomass [1]. The numbers of organisms capable of decomposing or adapting to decomposing pesticides could be increased by adding other suitable carbon sources to the soil [2]. Bacteria are important components of the soil microflora, as they are responsible for diverse metabolic processes that affect soil, such as nutrient cycling, organic matter formation and decomposition, soil structure formation, and biological control of insects, pathogens and weeds [3]. Fungi play a key role in ecosystem functioning; some are major decomposers of plant residues, releasing nutrients that sustain and stimulate plant growth in the process [4,5]. However, some fungi are soil-borne pathogens that cause root disease, such as the common fungal pathogen *Fusarium oxysporum* [6], which is a filamentous fungus that is widely distributed in the soil, water, subterranean and aerial plant parts, plant debris and other organic substrates [7]. These soil-borne fungi affect a wide variety of hosts of any age and generally produce symptoms such as wilting, chlorosis, necrosis, premature leaf drop, browning of the vascular system, stunting, and damping-off [8].

Soil-borne diseases are difficult to control. Various modes of action have been reported to control soil pathogens, including substrate competition, the ability to colonize the ecological niche favored by the pathogen, antagonism by antibiotics [9,10] or cell-wall degrading enzymes [11,12]. β-1,3-glucanase and chitinase secreted by mycoparasites, termites and bacteria are involved in the biological control activity against fungal plant pathogens [13–15].

**Fig 1:**
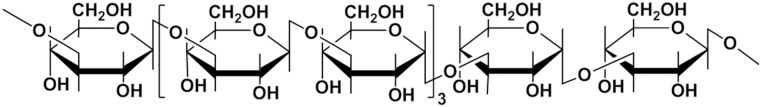
The chemical structure of the repeated unit of salecan.

Changes in the microbial community composition are often observed after the addition of organic or inorganic amendments [16], and different types of amendments may differ in organic matter composition (e.g., C/N ratio), which in turn, affects the decomposition rate and can change the microbial community structure. Salecan is a water-soluble and stable exopolysaccharide, which was extracted from the fermentation broth of *Agrobacterium* sp. ZX09 using centrifugation and ethanol precipitation as previously described [17,18]. Its structure consists of the following repeating unit: →3)-β-D-Glcp-(1→3)-[β-D-Glcp-(1→3)-β-D-Glcp-(1→3)]3-α-D-Glcp-(1→3)-α-D-Glcp-(1→ (Fig 1) [17]. The application of exopolysaccharide has been limited because of its properties of insolubility in aqueous media with physiological pH. While salecan has excellent biological activities and has been utilized in the food, medical and industrial fields [19–25]. In this study, we aimed to evaluate the effects of salecan on the composition of the soil microflora. The compositional shifts in the community structure of the soil microflora after applying salecan to vegetable patch soil were assessed using dilution plating, PCR-DGGE and quantitative PCR.

## Materials and Methods

### Experimental design

Soil samples were taken from a vegetable patch surface in Nanjing that belonged to Nanjing University of Science and Technology. The soil studies did not involve endangered or protected species; therefore, no specific permits were required for the described field studies. The plants in the vegetable patch were wilting. First removing the visible plant tissue in the soil, then dividing the soil into three groups, with three replicates which were from the same vegetable patch in each group. Each soil sample contained 5 kg of fresh soil; the first group was untreated, but 0.02% and 0.2% extracellular polysaccharide salecan was added to the second and third groups, respectively. Salecan was extracted from the fermentation broth of *Agrobacterium* sp. ZX09 by centrifugation and ethanol precipitation as previously described [17]. Commercial salecan (chemical compositions: sugar 77.13%, protein 6.2%, moisture 5.2%, and ash 10.28%; average molecular weight: 2×10^6^, water-soluble) was purchased from Karroten Company (Nanjing, China) [24]. The samples were watered once every two days to ensure that the water content was maintained at 50% the maximum water holding capacity. Soil samples were collected at 20, 40, 50 and 60 days after the application of salecan.

### Enumeration of culturable microorganisms

The population density of the cultivable bacteria in the soil was evaluated using serial dilution plating. The serial dilutions were prepared from a suspension of 1 g soil in 10 ml sterile phosphate buffer (15 mM, pH 7.0) by fully shaking the samples to disperse the soil in water, placing the samples on the shaking table for 30 min, and then diluting the samples to different concentration gradients. Appropriate dilutions were plated in triplicate on plates containing lysogeny broth agar (tryptone 10 g/l, yeast extract 5 g/l, NaCl 10 g/l, agar 15–20 g/l, pH = 7.0) or potato dextrose agar (peeled potatoes 200 g, glucose 20 g, agar 20 g, water 1000 ml, pH natural). Colonies were enumerated after 24 h and 48 h; total culturable bacterial counts were quantified by plating the serial dilutions on lysogeny broth agar medium, while the fungi were quantified by the plating on potato dextrose agar medium.

### Total DNA extraction from soil

Total DNA was extracted from 0.3–0.4 g of sample using a Soil Total DNA Extraction kit (Karroten, China). The quality of the extracted DNA was checked using horizontal electrophoresis on a 0.75% agarose gel, subsequent ethidium bromide staining and visualization under UV light.

### PCR amplification

Soil DNA was amplified using the 16S rDNA V3 primers 357F-GC and 518R (Table 1) [26] and ‘touchdown’ PCR to reduce the formation of spurious by-products [27]. The temperature profile for the PCRs was 5 min at 95°C, followed by 20 cycles of 30 s at 94°C, 30 s at annealing temperatures, 30 s at 72°C, and 10 cycles of 30 s at 94°C, 30 s at 55°C, and 30 s at 72°C. A final extension step was carried out for 10 min at 72°C, after which the DNA was stored at 4°C. The annealing temperature was decreased from the first cycle of 65°C to the last cycle of 55°C by 0.5°C every cycle. The fungal diversities in the soil were determined by DGGE analysis using the primers GC-Fung and NS1 (Table 1) [28]. The PCR program included an initial denaturation at 94°C for 5 min, followed by 30 cycles of 30 s at 94°C, 30 s of annealing at 55°C, and 30 s extension at 72°C. The final extension was 10 min at 72°C. The products were visualized by electrophoresis in 2% (w/v) agarose gels and ethidium bromide staining.

**Table 1:** PCR primers and targeted microorganisms used in this study.

Primer name	Orientation	Nucleotide sequence (5’–3’)	Targeted organism(s)
**357f** ^**a**^	forward	CCTACGGGAGGCAGCAG	Bacteria
**518r**	reverse	ATTACCGCGGCTGCTGG	Bacteria
**Fung** ^**a**^	forward	ATTCCCCGTTACCCGTTG	Fungi
**NS1**	reverse	GTAGTCATATGCTTGTCTC	Fungi
**ITS1-s3** ^**b**^	forward	CAGCGGAGGGATCATTACC	*Fusarium oxysporum*
**ITS1-as2OP** ^**b**^	reverse	CAAGAGATCCGTTGTTGAAAG	*Fusarium oxysporum*
**uTf** ^**b**^	forward	AACGTTACCAAACTGTTG	*Trichoderma* spp.
**uTr** ^**b**^	reverse	AAGTTCAGCGGGTATTCCT	*Trichoderma* spp.

^a^ Primers with a 40-bp GC clamp at the 5’ end.

^b^ Q-PCR primers.

### DGGE

DGGE analysis was performed using a Mutation Detection System (Bio-Rad, USA). Samples of the bacterial PCR product (40 μl) were loaded onto 10% polyacrylamide gels (acrylamide:bis-acrylamide, 39:1) in 1×TAE (4.84 g Tris base, 1.14 ml acetic acid, 2 ml 0.5 M ethylene diamine tetraacetic acid, in 1000 ml water, adjusted to pH 8.2). The gels consisted of a denaturing gradient ranging from 40% (at the top of the gel) to 60% (at the bottom); 100% denaturant was defined as a mixture of 7 M urea and 40% deionized formamide. Gels were run at 80 V at 60°C in 0.5×TAE buffer for 15 h. The fungal PCR products were separated by electrophoresis in 8% polyacrylamide gels consisting of a denaturing gradient from 25% to 40% in 1×TAE buffer at 60°C and 60 V for 16 h. After electrophoresis, the gels were stained with SYBR Green I (Invitrogen, USA) and photographed on a UV transilluminator (Bio-Rad, USA). The different DGGE bands from the samples were eluted from the gels by excising them from the UV-illuminated acrylamide gels and incubating them in 10 μl ddH_2_O at 4°C overnight. The eluted DNA was used for PCR amplification using primers without a GC-clamp. The PCR products were purified using a gel Extraction kit (Axygen, China) and cloned into pUM19-T (Vazyme, China) for sequencing. All the sequences in this study had been deposited in the GenBank nucleotide sequence database under accession numbers from KM110950 to KM110961.

### Q-PCR conditions

The real-time quantitative polymerase chain reaction (Q-PCR) of the total DNA sample was carried out on a real-time PCR system in a 20-μl reaction volume containing 1 × SYBR Green PCR master mix (Applied Biosystems, USA), 0.5 μM of each primer [29,30] (Table 1), and 75 ng of sample template. Amplification was performed using the real-time detection system and the following program: an initial denaturing step of 95°C for 5 min and then 45 cycles of denaturation at 95°C for 30 s, annealing at 55°C for 30 s and extension at 72°C for 30 s.

### Measuring β-1,3-Glucanase activity in the soil

β-1,3-Glucanase activity was assayed using laminarin as a substrate [31]. The glucose equivalents released from the enzyme reactions were determined colorimetrically using the dinitrosalicylic acid method [32], and the soil β-1,3-glucanase activity was defined as the amount of enzyme required to release 1 milligram glucose per gram soil per day (mg/g/d).

### Statistical analysis

The compositional shifts of the bacterial communities were assessed by comparing the DGGE banding patterns of replicate salecan-treated samples to those of the control soils. The scanned banding patterns of the DGGE profiles were analyzed using Quantity One gel analysis software (Bio-Rad) to generate similarity dendrograms via the unweighted pair-group method using arithmetic averages (UPGMA) clustering method [33]. The results are expressed as the means ± standard deviation (SD). A *P*-value less than 0.05 was considered a statistically significant difference.

## Results

### Effects of salecan on bacterial communities in the soil

**Fig 2:**
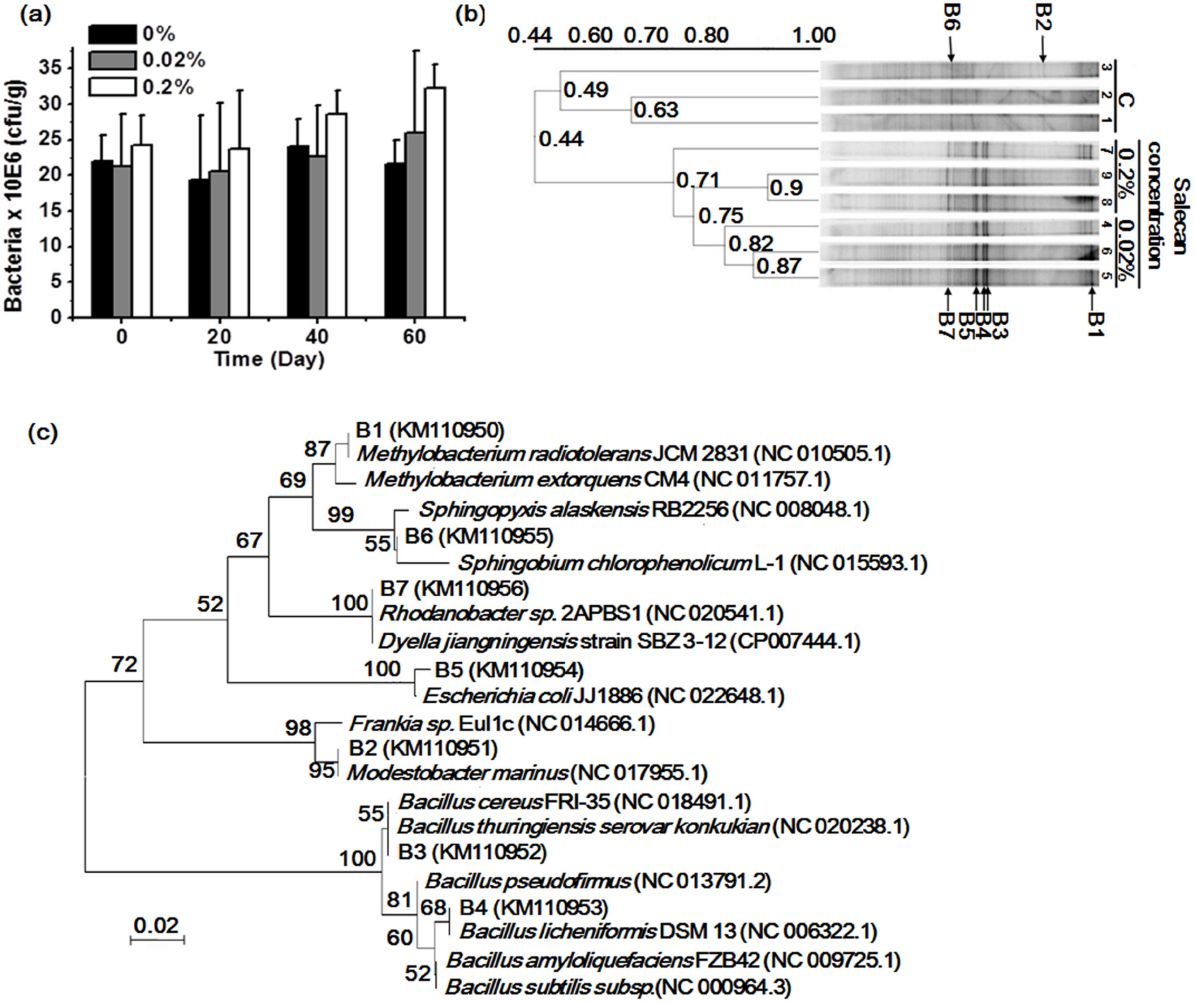
Changes in soil bacteria community structure. (a) Bacterial colony-forming units assessed using the culture plating method. All values are the means ± SD (n = 3). (b) Similarity dendrogram of the bacterial banding patterns of soil samples. The scale for 0.44–1.00 was the similarity coefficient among the samples. DGGE fingerprints of 16S rDNA sequences amplified using the primer pair 357fGC/518r from soil-extracted community DNA. Lanes 1, 2 and 3 show the results of soil samples without salecan treatment (C); lanes 4, 5 and 6 show the results of 0.02% salecan-treated samples; and lanes 7, 8 and 9 show the results of 0.2% salecan-treated samples. Seven changed DGGE bands (B1, B2, B3, B4, B5, B6, B7) are marked by arrows. (c) The phylogenetic relationship between the 16S rRNA gene sequences of the soil samples and the related organisms from the GenBank database. The dendrogram was generated using the neighbor-joining method, with 1000 bootstrap resamplings. The scale bar represented 0.02, estimated nucleotide changes per sequence position.

Serial dilution and plating were used to determine the colony-forming units (S1 Table). As shown in Fig 2a, there was no significant difference between the soil samples treated with 0.02% or 0.2% salecan and the group without treatment from the 20th to 60th days. DGGE using 16S rDNA revealed complex banding patterns and indicated distinct shifts in the bacterial community structure of each soil sample on 60th day (Fig 2b). The soil samples were from the same date for bacterial communities. The DGGE results revealed significant changes between the control and salecan groups. UPGMA clustering of the DGGE data indicated that the bacterial community structure differed among the 9 soil samples; the samples could be divided into 2 groups: with and without salecan. Although most of the samples treated with 0.02% or 0.2% salecan clustered into one group, the samples treated with 0.02% salecan also clustered into a subgroup separate from the 0.2% salecan-treated samples. Through observing the DGGE pattern, seven bands (B1, B2, B3, B4, B5, B6 and B7) were selected for further sequencing analysis because they showed the most significant changes among the three groups. Sequence analysis of the excised bands gave insight into the dominant taxa of the microbial succession. Dendrograms were constructed to visualize the relationships between the sequences from the soil samples and related organisms from the GenBank database (Fig 2c). The 7 bacterial bands were classified into one *Bacillus* group and 2 other groups based on the 16S rDNA gene sequence analysis.

### The effects of salecan on the fungal community

**Fig 3:**
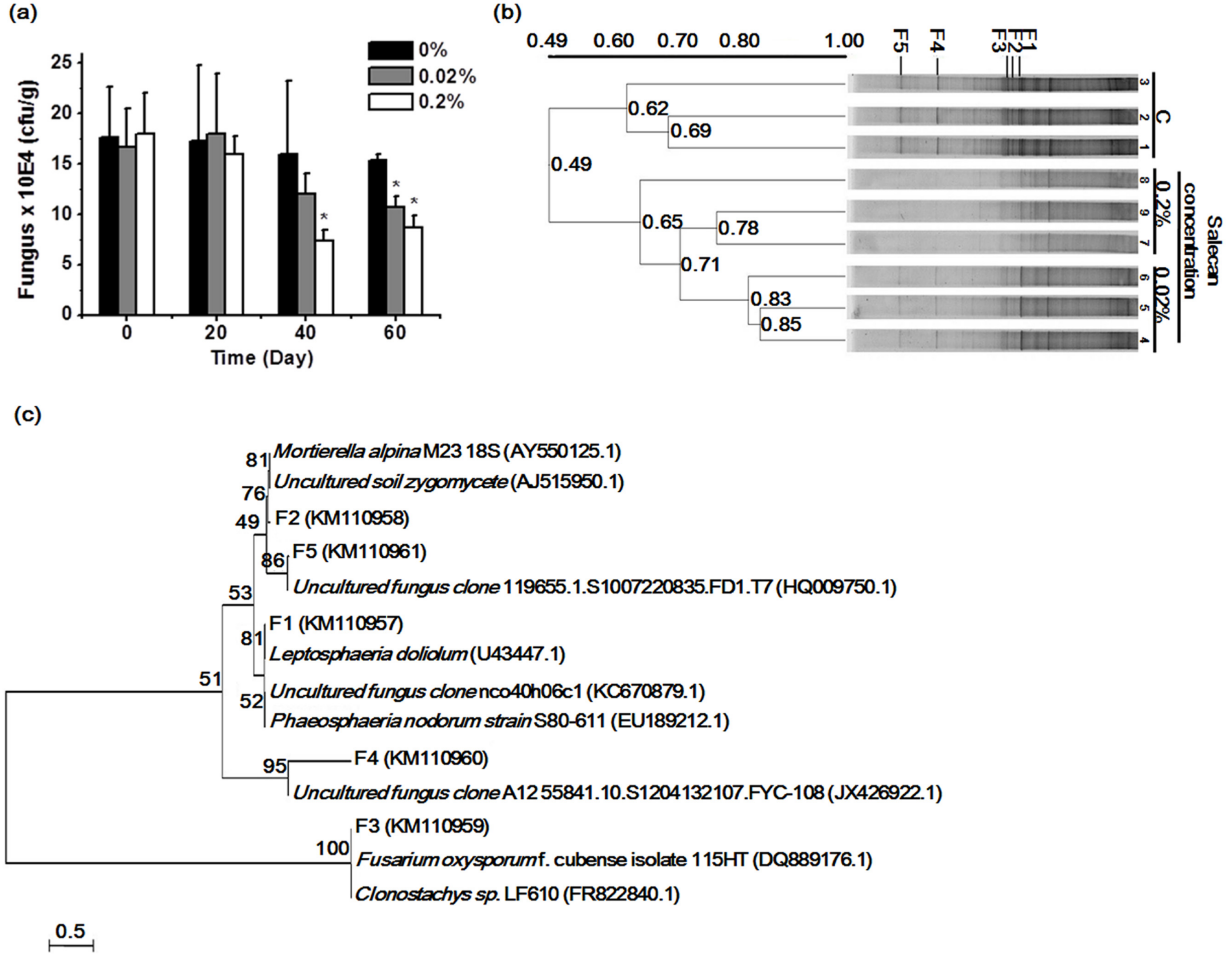
Changes in the soil fungus community structure. (a) Fungal colony-forming units assessed using the culture plating method. All values are the means ± SD (n = 3). *P < 0.05 vs 0% group. (b) Similarity dendrogram of the fungal banding patterns of the soil samples. The scale for 0.49–1.00 was the similarity coefficient among the samples. DGGE fingerprints of the 18S rDNA sequences amplified using the primer pair Fung-GC/NS1 from soil-extracted community DNA. Lanes 1, 2 and 3 show the results of soil samples treated without salecan (C); lanes 4, 5 and 6 show the results of 0.02% salecan-treated samples; and lanes 7, 8 and 9 show the results of 0.2% salecan-treated samples. Five changed DGGE bands (F1, F2, F3, F4, F5) are marked by vertical black lines. (c) The phylogenetic relationship between the 18S rRNA gene sequences from the soil samples and related organisms from the GenBank database. The dendrogram was generated using the neighbor-joining method with 1000 bootstrap resamplings. The scale bar represented 0.5, estimated nucleotide changes per sequence position.

Soil samples were diluted and plated on potato dextrose agar medium to analyze the fungi (S2 Table). On the 40th and 60th days, the fungal counts were significantly decreased in the soil samples treated with 0.02% or 0.2% salecan (Fig 3a). The 18S rDNA DGGE analysis revealed complex banding patterns, indicating distinct shifts in the fungal community structure of each soil sample on the 60th day (Fig 3b). The soil samples were from the same date for fungal communities. Clustering the DGGE data revealed that the fungal community structure differed among the 9 soil samples, and all samples could be divided into 2 groups: with and without salecan. Five bands (F1, F2, F3, F4 and F5) were selected for further sequencing analysis because they showed the most significant changes among the three groups.

A dendrogram was constructed to visualize the relationships between the sequences of the soil samples and related organisms from the GenBank database (Fig 3c). The 5 fungal bands were classified into 4 groups, and among these 4 groups, the F1 and F3 bands were classified into 2 different groups and had a close genetic relationship with two pathogenic fungi, *Phaeosphaeria nodorum* and *F*. *oxysporum*.

### Detection and quantification of soil-borne fungi

**Fig 4:**
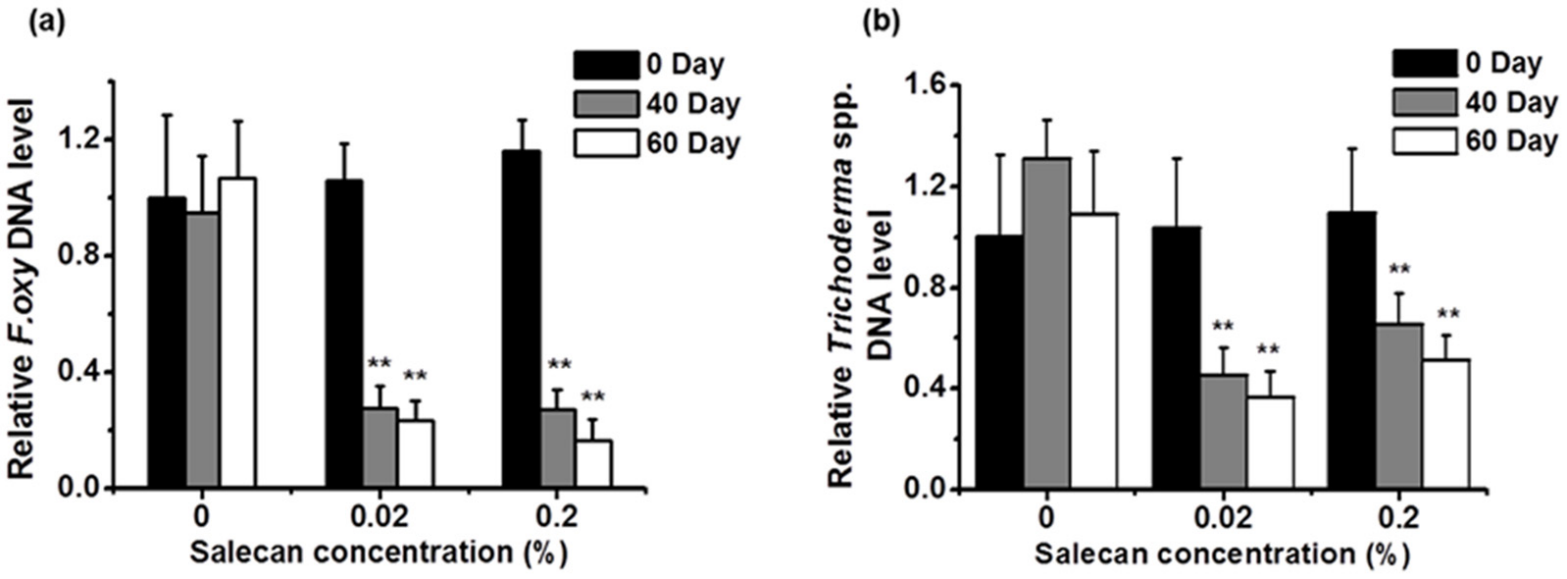
Quantification of soil fungi. The relative abundances of the two fungal groups in soil samples treated with different concentrations of salecan, as estimated using qPCR assays. The results show DNA from *Fusarium oxysporum* (a) and *Trichoderma* spp. (b). All values are the means ± SD (n = 3). **P < 0.01 vs 0% group.

As an alternative to DGGE for tracking microorganisms, Q-PCR can analyze the total DNA of a specific microbe extracted from soil samples. Based on the 18S rDNA DGGE band sequence analysis, the F3 band, which was identical to the 18S rDNA of *F*. *oxysporum*, was absent from the samples inoculated with salecan; therefore, we used the fungal ITS1 genes to detect and quantify *F*. *oxysporum* and *Trichoderma* spp. using Q-PCR assays (S3 Table) [29,30]. As shown in Fig 4, treatments with 0.02% or 0.2% salecan for 40 or 60 days resulted in a significantly decreased amount of *F*. *oxysporum* and *Trichoderma* spp. DNA compared with the without-salecan-treatment group (0%). These results showed that salecan treatment decreases the amount of *F*. *oxysporum* and *Trichoderma* spp. fungi in the soil, which agreed with our findings using the DGGE method.

### β-1,3-Glucanase activity in salecan-treated soil

**Fig 5:**
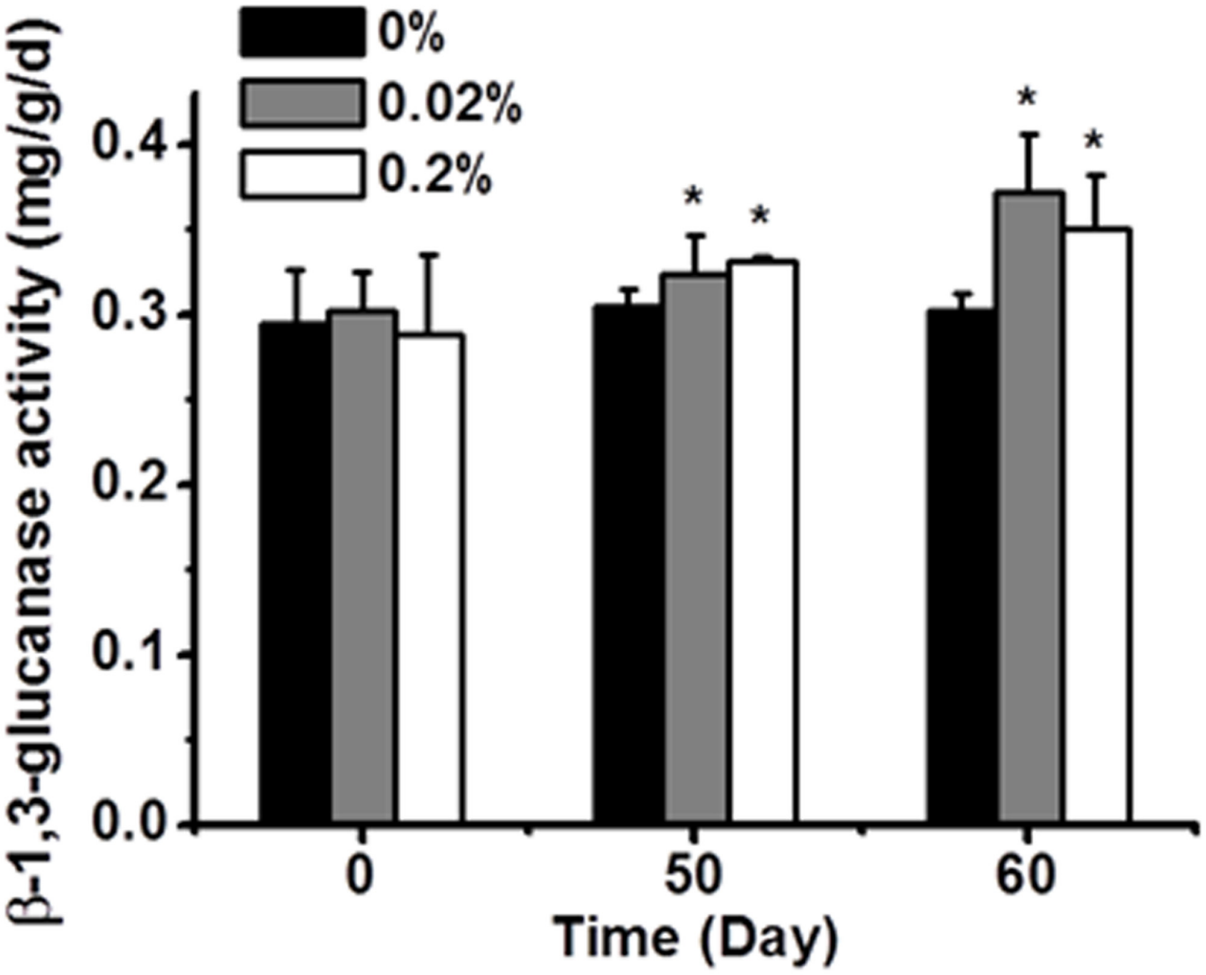
β-1,3-Glucanase activity in soil samples treated with different amounts of salecan. The soil β-1,3-glucanase activity was defined as the amount of enzyme required to release 1 mg of glucose per day per gram soil (mg/d/g). All values are the means ± SD (n = 3). *P < 0.05 vs 0% group.

The secretion of extracellular enzymes capable of lysing cell walls of pathogenic fungi is important for parasitic control processes, and these enzymes are well characterized in many biocontrol agents. Therefore, the glucanase activity was measured in the soil after treatment with salecan (S4 Table). On the 50th and 60th days, the β-1,3-glucanase activities in soil inoculated with either 0.02% or 0.2% salecan were significantly increased compared with the control group (Fig 5).

## Discussion

Soil is a complex microbial habitat, and it is not surprising that large shifts in carbon sources in soil may affect the activity, ecology and population dynamics of the microbes [34]. In our previous studies, we showed that salecan consumption changed the microbiota composition in mouse cecum [24]. The present work aimed to investigate the effects of salecan on the population dynamics of native soil microbial communities. We showed that salecan consumption changed the microbiota composition and increased the β-1,3-glucanase activities in the soil.

Sole carbon source tests, which were designed to identify microbial isolates, can be used to metabolically fingerprint soil microbial communities; however, the carbon source profiles were not selected for this study [35,36]. On the other hand, the soil microbial community distribution is different upon addition of different carbon sources. In our study, salecan was added to the soil to serve as a carbon source, which increased the amount of *Bacillus* spp. and β-1,3-glucanase activity. We did not observe the changes of soil chemical and physical properties upon addition of these amounts of salecan. There was also no literature pointing out that the low concentration of exopolysaccharide can influence the soil physical and chemical properties. *Bacillus* spp., such as *B*. *halodurans*, *B*. *subtilis*, *B*. *brevis*, *B*. *licheniformis*, and *B*. *amyloliquefaciens* [37–41], can produce diverse extracellular polysaccharide-degrading enzymes [42]. Additional carbon source and nutrients can help microbes survive and colonize [43,44], and thus, their population could be increased by the addition of salecan. A wide range of prokaryotic and eukaryotic microorganisms have the potential to produce cell-wall-degrading enzymes when glucan, chitin or isolated fungal cell wall material are present in the growth medium [45,46]. A similar study reported that the chitinase and glucanase activities of *Clonostachys rosea* were induced by fungal cell wall extracts or by polymers such as laminarin and chitin [13].

We tested the direct inhibition of salecan (form 0.2 to 2%) on growth of *F*. *oxysporum*, and found no inhibition (data not shown). The decrease in fungi can be explained by the increased activity of β-1,3-glucanase and increased *Bacillus* population. Fungal cell walls consist of four main components: glucan, chitin, mannan and mannoprotein [47–50]. Glucans contained β-1,3-glucan, mixed β-1,3-/β-1,4-glucan, β-1,6-glucan, and α-1,3-glucan [51]. Glucans are not only the cell wall components, but also could function as true virulence factors [52]. Such as when α-1,3-glucan is injected alone into the mice, it stimulates the host immune response and consequently has an antifumigatus development role. β-1,3-glucanases and chitinases inhibit spore germination and have lytic activity against the cell walls of a number of plant pathogenic fungi, including *Botrytis cinerea*, *Rhizoctonia solani*, *Sclerotium rolfsii*, and *F*. *oxysporum* [53–56]. *Bacillus* spp. produce diverse extracellular polysaccharide-degrading enzymes and are able to produce several broad-spectrum antibiotics and exhibit antagonistic behavior against fungal pathogens through competition or exploitation [57–59]. Helistö et al. have reported that *Bacillus* sp. X-b, a biocontrol agent against certain plant pathogenic fungi, secretes a complex of hydrolytic enzymes, composed of chitinase, chitosanase, laminarinase, lipase and protease [60]. And Leelasuphakul et al. have also demonstrated that crude extracts and β-glucanase produced by *Bacillus subtilis* had a synergistic effect on the inhibition of fungal growth with secondary metabolites and against the green mold pathogen of citrus fruit [61,62].

As bacilli are effective against a wide range of fungal pathogens, it’s tempting to speculate on the actual antifungal mechanisms. One factor is the fast bacterial growth rate in the presence of various carbon sources and the ability to compete for nutritional resources, especially in poor soil habitats. This may be accompanied by the activity of hydrolyzing enzymes such as chitinase and β-1,3-glucanase, which not only degrade fungal cell walls liberating carbon source but also cause mechanical damage to the fungal structures. It is evident that chitinolytic and glucanolytic enzymes play a major role in antifungal activity [63,64].

Microorganisms fulfill important ecosystem functions for plants and soils. They affect the plant health and growth, enhance stress tolerance, provide disease resistance, aid nutrient availability and uptake and promote biodiversity [65,66]. Plant growth promotion by microbes was based on improved nutrient acquisition, hormonal stimulation, lipo-chito-oligosaccharides, and biocontrol of pathogens by inducing systemic resistance in the host plant and increasing root adhering soil by exopolysaccarides [67]. Members of the bacterial genera *Azospirillum* and *Rhizobium* are well-studied examples for plant growth promotion, *Bacillus*, *Pseudomonas*, *Serratia*, *Stenotrophomonas*, and *Streptomyces* are model organisms to demonstrate influence on plant health [68].

The role of β-1,3-glucanase in preventing fungal disease has been well studied, for example, glucanases have been shown to serve as bio-control agents in agricultural applications by protecting plants from fungal invasion [69–71]. Bacteria secreted glucanase and other fungal cell wall-degrading enzymes are commonly considered as potential biocontrol agents against plant-pathogenic fungi [72–74]. Therefore, degrading enzymes or the bacteria that produce these enzymes could be used as biological control agents [15,75]. Many chitinolytic and glucanolytic microorganisms have potential for controlling fungal plant pathogens, but they are not fully successful in all cases due to different geological and environmental conditions. Thus, we tested the idea of whether the β-glucan salecan can increase the numbers of biological-control bacteria and decrease the amount pathogenic fungi in soil.

## Conclusions

The data presented in this study clearly demonstrate that salecan reshapes the bacterial and fungal communities and increases the β-1,3-glucanase activity in soil samples, causing significant decreases in the soil-borne fungi *F*. *oxysporum* and *Trichoderma* spp. Therefore, salecan has the potential to be applied in agriculture to prevent and control soil-borne pathogenic fungi.

## Supporting Information

S1 Table The population density of the cultivable bacteria in the soil. (XLSX)

S2 Table The population density of the cultivable fungi in the soil. (XLSX)

S3 Table Detection and quantification of soil-borne fungi. (XLSX)

S4 Table β-1,3-Glucanase activity. (XLSX)
